# Fenxiang biota: a new Early Ordovician shallow-water fauna with soft-part preservation from China

**DOI:** 10.1007/s11434-015-0762-7

**Published:** 2015-03-17

**Authors:** Andrzej Balinski, Yuanlin Sun

**Affiliations:** 1 Instytut Paleobiologii PAN, Twarda 51/55, 00-818 Warszawa, Poland; 2 Key Laboratory of Orogenic Belts and Crustal Evolution, School of Earth and Space Sciences, Peking University, Beijing, 100871 China

**Keywords:** Antipatharia, Brachiopoda, Hydrozoa, Nematoda, Embryos, Problematica, 黑珊瑚, 腕足动物, 水螅虫, 线虫, 化石胚胎, 分类位置不明化石

## Abstract

Our perception of biodiversity in the geological past is incomplete and biased because most organisms did not have mineralized skeletons and therefore had little chance of fossilization. This especially refers to shallow-water marine environments, rarely represented by localities with exceptional preservation of fossil material (known as taphonomic windows or Konservat-Lagerstätten). Such extraordinary “windows” may markedly broaden our knowledge of biodiversity of the past. Here, we show a review of the invertebrate fossils from recently discovered locality in the Lower Ordovician Fenxiang Formation of Hubei Province in southern China revealing exceptional preservation of soft tissues. The fauna, generally of shallow-water aspect, contains linguloid brachiopods with a remarkably preserved pedicle, the oldest traces of nematode life activities, the oldest reliable record of hydroids, the first fossil antipatharian corals, a pyritized colonial organism of unknown affinity, supposed arthropod appendages, probable phosphatized scalidophoran worm embryo and other fossils. Our discovery supports the opinion that the famous soft-bodied preservation of Burgess Shale- or Chengjiang-type did not vanish from the fossil record in post-Cambrian times. The new finding represents a prelude to the Great Ordovician Biodiversification Event and provides evidence for calibration of molecular clock of several invertebrate lineages.

## 抽象

以有软躯体保存而闻名的伯吉斯页岩型或澄江型化石生物群的发现,为我们打开了一扇全面认识地质历史中生物多样性的窗口。在湖北宜昌地区新发现的早奥陶世分乡生物群就是这样的一个窗口。该动物群包括了保存有肉茎结构的舌形贝类、最早的线虫生活痕迹、最早而可靠的水螅虫化石记录、最早的黑珊瑚化石记录、一种亲缘关系不明群体生物化石、节肢动物附肢化石和可疑的scalidophoran蠕虫胚胎化石等。同时新发现为若干无脊椎动物类群分子钟的标定提供了直接的化石证据。

## Introduction

**Fig. 1:**
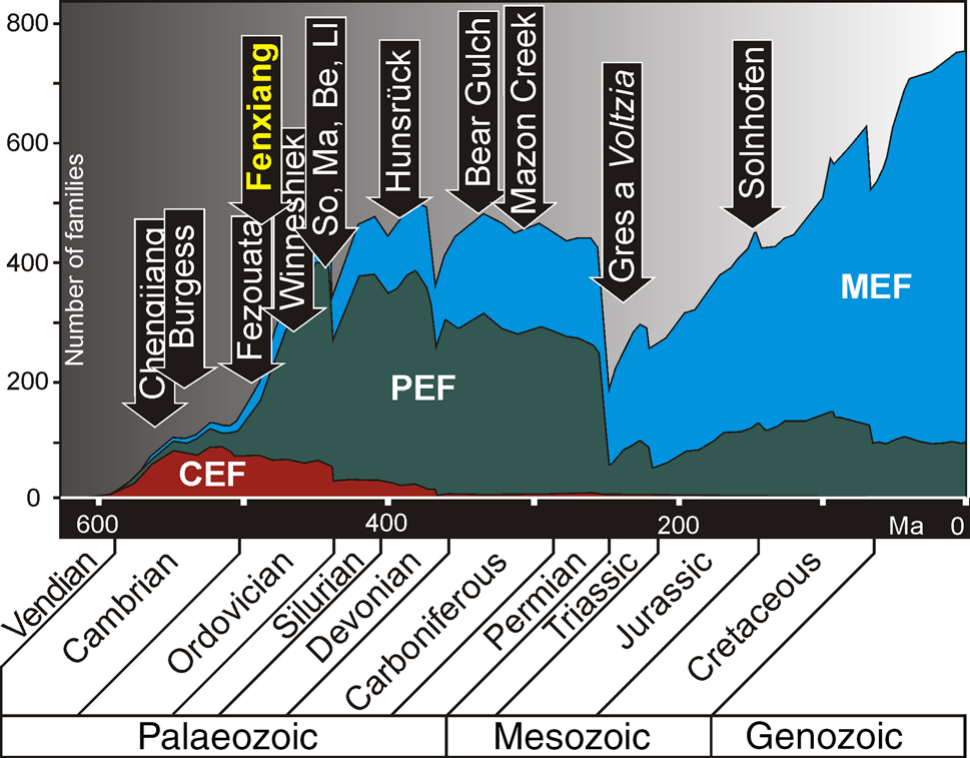
Occurrence of the most important soft-bodied faunas on the background of the family-level diversity of marine animals through the Phanerozoic with three great Evolutionary Faunas denoted; CEF: Cambrian EF, PEF: Paleozoic EF, MEF: Modern EF, modified after Sepkoski [40]. Other abbreviations: So, Soom Shale; Ma, Manitoba; Be, Beecher’s Trilobite Beds; Ll, Llanfawr Mudstone (more explanation in the text)

**Fig. 2:**
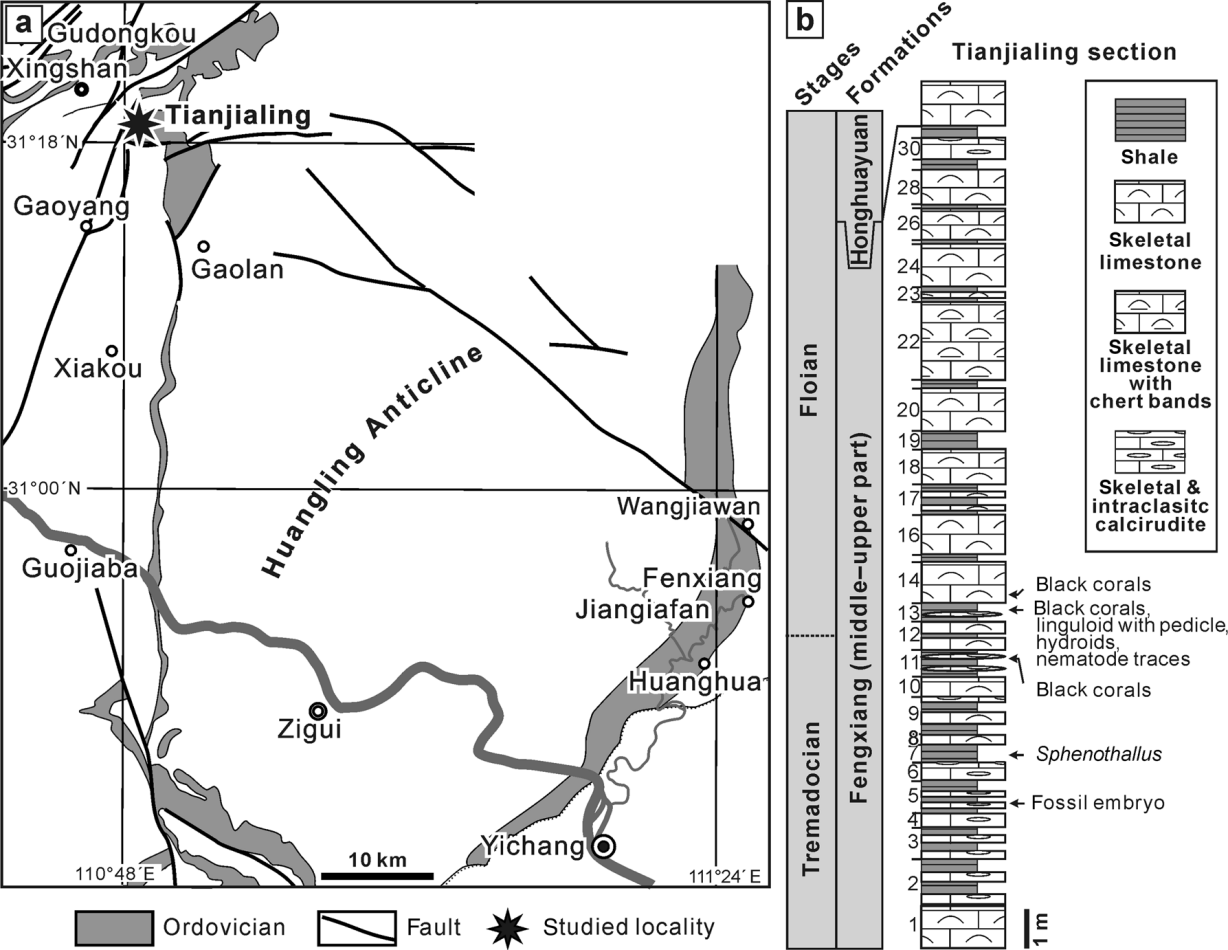
Simplified locality map of the exceptionally preserved Early Ordovician fossil fauna (**a**) and rock column of the Fenxiang Formation, (**b**) near Tianjialing, Hubei Province

The classic examples of taphonomic windows that have been essential for exploring the earliest Paleozoic biodiversity and phylogenetic metazoan differentiation are the Early Cambrian Chengjiang (Yunnan Province, China) and the Mid-Cambrian Burgess Shale (British Columbia, Canada) soft-bodied biotas [1–3]. Until the last decade, it was believed that these renowned Cambrian soft-bodied faunas vanished from the fossil record well before the end of the Cambrian [4]. In recent years, however, some abundant and well-preserved Burgess Shale-type (BST) faunas have been also found in post-Cambrian strata [4–8]. Although Ordovician faunas with preserved soft parts are still remarkably rare globally [5, 7], they indicate that the BST soft-bodied faunas, on one hand, and specific taphonomic conditions, on the other, persisted sporadically beyond the Cambrian [8]. Examples of such remarkable preservation in the Ordovician are soft-bodied or lightly sclerotized assemblages in the Tremadocian (Early Ordovician) Fezouata Formation in Morocco [4], the Late Ordovician of New York State (Beecher’s Trilobite Bed) [5], the Late Ordovician of Manitoba [6] and Llanfawr Mudstone of Wales [7], Middle Ordovician Winneshiek Lagerstätte in northeast Iowa [9], and the Hirnantian (Late Ordovician) Soom Shale of South Africa [10] (Fig. 1). These strata record mostly environments similar to their Cambrian counterparts which are open-marine setting with anoxic bottom waters (Fezouta Formation) [4], or in deep dysoxic bottom waters with low diversity fauna (Beecher’s Trilobite Bed) [5]. Others were deposited in cool, shallow marine environments with intermittent anoxia (Soom Shale) [11]. Evidence from open-marine, shallow-water faunas, which are equally essential for study of the Early Ordovician biodiversification, remains poorly represented in the fossil record. Recently discovered fauna from the Lower Ordovician Fenxiang (transcribed also Fenhsiang) Formation exposed at Tianjialing in Three Gorges area of Hubei Province (Fig. 2) may fill, at least partially, this gap. The fauna from the Fenxiang Formation represents preliminary stage to the Great Ordovician Biodiversification Event (GOBE). Here, we present a short overview of this fauna including both recently reported or hitherto undescribed fossils.

## Geological overview

The presented material was collected for the most part from the middle part of the Fenxiang Formation at Tianjialing village (Fig. 2) during several field trips between 2008 and 2014. The investigated area is located at the center of the Yangtze Platform, which during Ordovician was covered by well-defined, continuous sediment sequence composed of shelly limestone and graptolite shale facies. The sequence is widely exposed around the Huangling Anticline in the Three Georges area of Hubei Province [12]. The Fenxiang Formation is ca. 40 m thick in the east and ca. 20 m thick in the west of the anticline, mainly consisting of black to greenish gray shale intercalated with dark gray to gray skeletal and peloidal limestones and containing abundant graptolites and various shelly fossils including brachiopods, bryozoans, trilobites, cephalopods, etc. [13]. The rock and fossil characteristics suggest frequent changes in the sedimentary setting from quite deep-water basin to energetic shallow-water carbonate platform. Although the Fenxiang Formation was commonly considered as being of mainly late Tremadocian age, the occurrence of the conodont *Acodus triangularis* in Bed 13 of the studied section indicates that the upper part of the formation is of early Floian (Arenig) age [14, 15].

**Fig. 3:**
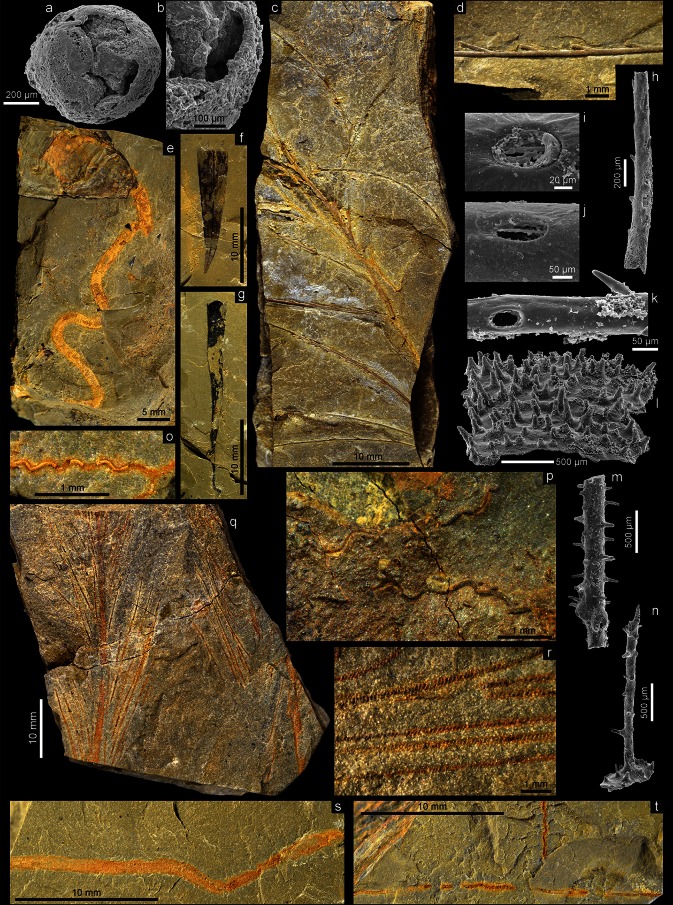
Exceptionally preserved Early Ordovician fauna from the Fenxiang (**a**–**o**, **q**–**t**) and Dawan (**p**) formations, Yichang area, Hubei Province, China. **a, b** Spheroidal microfossil showing similarity to fossilized scalidophoran embryo *Markuelia* Val’kov 1984 at cleave stage with preserved blastomeres, in general view (**a**) and enlargement showing details of thin external membrane (**b**), **c, d** Pyritized colony of hydroid *Sinobryon elongatum* Baliński, Sun and Dzik 2014 (**c**) and enlargement of lateral branch showing elongate zooids (**d**), **e** Linguloid brachiopod *Leontiella* sp. with preserved pyritized vermiform pedicle; see also Baliński and Sun [16], new photograph, **f** Nearly complete conulariid specimen from the Fenxiang Formation at Jiangiafan, **g** Partially decorticated specimen of *Sphenothallus* sp., **h**–**k**) Arthropod appendages showing preserved setae (**h**, **k**) and setal sockets (**i**, **j**); possible longitudinal filaments of phosphatized infesting bacteria visible in the setal sockets (**i**, **j**), **l**–**k** Phosphatic coralla of antipatharian coral *Sinopathes reptans* Baliński, Sun and Dzik 2014 showing basal part of a colony with crowded spines (**l**), spinose branch (**m**), and partly preserved spinose basal part of colony and erect stem (**n**), **o**, **p** Long sinusoidal nematode tracks from Fenxiang (**o**) and Dawan (**p**) formations, **q**, **r** Pyritized colony of an enigmatic organism in general view (**q**) and enlargement of lateral branches (**r**), **s**, **t** Examples of trace fossils on split surfaces

Among fossils occurring in the formation, bryozoans, trilobites, orthide brachiopods, nautiloids, bivalves, and benthic graptolites are the most common [16]. Generally, the fossils are rather sparse and usually fragmented, but occasionally well-preserved specimens revealing the preservation of soft tissues are also present. Among the most important fossils from the Fenxiang Formation are linguloid brachiopods (Fig. 3e) preserving soft parts with remarkable fidelity [17], probably the oldest reliable, pyritized colony of hydroids (Fig. 3c, d) [18], pyritized horizontal burrows which represent the oldest record of life activity of marine nematodes (Fig. 3o) [19], and a pyritized colonial organism of unknown affinity (Fig. 3q, r). Generally, specimens with soft-part preservation are extremely rare in the formation. Among the skeletonized fauna the discovery of remnants of fossil antipatharian corals (Fig. 3l–n) is noteworthy as they were previously unknown in fossil record [14]. Finally, the findings of microscopic soft organisms preserved by phosphate encrustation or replacement (tubular arthropod appendages and probable scalidophoran embryo) in the limestone intercalations suggest that the three-dimensional “Orsten”-type preservation also played some role in the taphonomic processes of the Fenxiang fauna. The most important discoveries in the Fenxiang Formation are briefly commented on below.

Although the beds with described fauna were presumably deposited in open-marine, rather deep-water settings, several elements of the fauna exhibit an allochthonous shallow-water origin. These shallow-water forms were probably transported basin-wards by currents or occasional heavy storms from settings adjacent to reefal buildups. The buildups, described from the Fenxiang Formation at the Chenjiahe section, are the oldest known bryozoan reefs (constructed mainly by bryozoans, lithistid sponges, pelmatozoans, stromatoporoids, and calcimicrobes), which reflect the initiation of the GOBE [20].

All specimens described and illustrated are housed at the Geological Museum of Peking University, Beijing, China (PKUM), and the Institute of Paleobiology, Polish Academy of Sciences, Warsaw, Poland (ZPAL).

## Results and discussion

The preserved soft parts in specimens from the Fenxiang Formation are of bright reddish-yellow to brown color resulting from the weathering and oxidation of pyrite that apparently precipitated on decaying carcasses shortly after their rapid burial in the sediment [17]. The framboidal pyrite structure is still well recognizable although at present, the framboids are pseudomorphed by clay minerals and iron oxide [17, 19]. The pyritized soft parts are preserved either slightly compressed or with their almost original three-dimensionality.

Besides the famous Cambrian BST faunas, the findings of linguloid brachiopods with preserved soft parts are extremely rare in the fossil record. The first finding of a linguloid (*Lingula*? *lesueuri*) with preservation of pedicle was reported by Davidson [21] more than a century ago from the Devonian of Devonshire, England. A few years later, Walcott [22] described *Lingula equalis* Hall with preserved pedicle from the Middle Ordovician of New York State. Both specimens, however, are quite poorly preserved and not very informative. Three linguloid specimens with unquestionable pyritized pedicle were described from the Lower Devonian Hunsrück Slate (Germany) [23]. The recent finding of a short cylindrical stalk interpreted as a pedicle of the discinoid *Kosoidea cedarbergensis* from the Upper Ordovician Soom Shale of South Africa is also noteworthy [10]. Under these circumstances, the present discovery from the Lower Ordovician Fenxiang Formation of the linguloid *Leontiella* with a preserved pedicle is of great importance (Fig. 3). This is the first record of preserved linguloid pedicle with remarkable fidelity in the post-Cambrian strata [17]. The recovered specimens show a three-dimensional, pyritized pedicle with detailed preservation of the external morphology (Fig. 3e). The long vermiform and flexible pedicle, as well as the elongated streamlined shell with a radial ornament (characteristic for burrowing linguloids), suggest that Early Ordovician *Leontiella* is one of the earliest linguloids which acquired an infaunal mode of life [17].

Infaunal activity is also recorded in the Fenxiang assemblage by variety of trace fossils. Besides large-sized macroscopic traces, the assemblage contains also numerous microscopic, cylindrical and almost regularly sinusoidal burrows of 20–60 µm in diameter (Fig. 3o). They are mostly horizontal and are entirely and tightly filled with weathered spherical framboids suggesting that the original mineral was pyrite. The framboidal pyrite most probably precipitated as a result of bacterial decay of the organic-rich original infilling of the burrows (e.g., fecal pellets) [19]. The geometry and dimensions of these sinusoidal traces from the Fenxiang Formation closely resemble burrows of modern marine, free-living nematodes. The Early Ordovician traces represent the oldest record of life activity of marine nematodes preceding the oldest known nematode body fossil (the herbivore *Palaeonema phyticum* from the Lower Devonian Rhynie chert from Scotland [24]) by around 70 Ma and the oldest nematode trace fossil (*Cochlichnus anguineus* from the Middle Triassic of Germany [25]) by around 230 Ma. Recently, a problematic large-sized nematode was described from the earliest Floian of the Xiayangao section in Guizhou Province, southern China [26]. During more recent investigations, trace fossils similar to those from the Fenxiang Formation at Tianjialing were also revealed from the coeval strata exposed at Huanghua near Yichang (about 90 km southeast of Tianjialing) and the Arenigian Dawan Formation exposed 7 km west of Tianjialing (Fig. 3p). The fossil material reveals that marine nematodes of a very similar size to the extant ones were already present in the Early Ordovician [19]. However, their mode of life and body plan probably developed no earlier than the mid-Early Cambrian due to unfavorable environmental conditions in the Ediacaran and earliest Cambrian [19]. Only after the Seilacher’s Agronomic Revolution, expressed mainly in heavy bioturbation of sediment [27] which destroyed extensive microbial mat covers [28, 29], did interstitial environments become habitable to nematodes.

Large-sized burrowing traces and coprolites in shale of the Fenxiang Formation are also present. These burrows are more linear than the nematode ones and measure 0.2–2.7 mm in diameter. The large-sized burrows are much more flattened, sometimes almost without relief, as a result of considerable compaction (Fig. 3s). However, the burrows measuring <1 mm in diameter are filled with secondary oxidized and pseudomorphed framboidal pyrite and thus preserve more three-dimensional aspect (Fig. 3t). All observed burrows are parallel to the bedding surface and filled with slightly darker and coarser sediment which shows slight enrichment in iron oxides (Fig. 3s, t). The trace fossils of the Fenxiang Formation, although rare, indicate that varied soft-mud dwellers were present.

The finding of an advanced hydroid with pyritized colony in the Fenxiang Formation is also significant (Fig. 3c, d). The specimen (part and counterpart), like other cases of preservation of soft tissue, is easily discernible on the rock surface due to the distinction between the weathered pyritized body and the sediment background. The revealed specimen is an upright monopodial colony with helically arranged side branches arising at an acute angle around the central rachis. The zooids are elongate fusiform and arise uniserially on the upper surface of branches (Fig. 3d). Although no thecae are preserved, it is probable that the Fenxiang form may represent some thecate hydroids, possibly members of Haleciidae [18]. There was a general opinion that the hydras and medusae are primitive organisms and they are expected to occur in old rocks. Critical review of the Precambrian and Cambrian hydrozoans have shown, however, that these forms represent other groups of invertebrates [18]. For example, several Ordovician and Silurian hydrozoan findings have recently been interpreted as graptolite stolons [30]. In this way, most if not all ancient hydrozoans have been erased from the list of the Early Paleozoic fossils. The oldest reliable medusae come from the Upper Carboniferous Mazon Creek fauna [31], and the oldest hydrozoan colony was found in the Upper Ordovician of Kentucky [32]. A possible pyritized solitary hydrozoan has been recently reported from the Upper Ordovician Llanfawr Mudstone of Wales [7].

The timing of appearance of coloniality in hydroids is crucial in calibration of the cnidarian molecular phylogenetic tree. Unfortunately, few fossil records are available for such calibration, hence the great importance of the present Chinese finding. The hydroid now recovered from the Fenxiang Formation implies that the main diversification of the group preceded the Floian.

The Fenxiang Formation contains rare organo-phosphatic conical fossils usually regarded as cnidarians representing an epifaunal suspension-feeding mode of life. One of them is represented by a single, well-preserved specimen belonging to conulariids (scyphozoan cnidarians). Its theca is smooth and measures 16.7 mm in length and 4.1 mm in width at the apertural end (Fig. 3f). The specimen shows great resemblance to ?Conulariid gen. et sp. indet. described recently from the Lower Ordovician Tonggao Formation of Guizhou Province, China [33]. Another conical fossil from the Fenxiang Formation, also represented by a single specimen, belongs to the cnidarian genus *Sphenothallus* Hall. The elongate, very slender theca measures 26.5 mm in length and 2.7 mm in width at the apertural end (Fig. 3g). It is weakly sinuously curved and reveals well-preserved apical part with holdfast. This form differs from all other species of the genus by the presence of long radiating spines developed at the basal part of the theca. This is the second finding of *Sphenothallus* in the Ordovician of China—recently the genus has been reported from the Lower Ordovician of Guizhou Province [33].

One of the most important discoveries in the Fenxiang fauna is the finding of the first fossil black corals (Antipatharia) (Fig. 3l–n). Despite their prevalence in modern coral faunas and importance in the recent marine ecosystems, they were unknown in the fossil record. Numerous phosphatic fragments revealed in carbonate samples from the Fenxiang Formation processed in acetic acid show astonishing similarity to skeletal elements of the extant antipatharians, despite the huge time interval (approximately 470 Ma) separating them [14]. The corallum of the Fenxiang form called *Sinopathes reptans* consists of an extensive encrusting basal part and the erect stem with lateral branches (Fig. 3l–n). Both the basal and erect parts of the corallum are covered with slender spines up to 600 µm in length. The discovery of antipatharians in the Lower Ordovician suggests that the main differentiation of the Hexacorallia clade should precede this date. This is in accordance with estimates inferred from the molecular clock calibrated with the scleractionian fossil record [34].

Among the non-biomineralized forms revealed in the Fenxiang assemblage, there are rare specimens of a colonial feather-like organism of uncertain affinity (Fig. 3q, r). It shows a strong and quite straight central rachis and numerous side branches arising from it at an acute angle. The branches are usually almost perfectly straight suggesting their apparent rigidity. However, the bent branches observed on one of the specimens suggest that they also possessed some elasticity (Fig. 3q, right specimen). Although superficially, the colony shows some similarity to the Wenlock–Famennian feather-like hydroid *Plumalina* [35] and Lower and Mid Ordovician possible hydroid *Webbyites* [36]. The relation of the Fenxiang fossil and its anatomy await detailed study.

Of special interest is the recovery of a single spherical phosphatized microfossil (Fig. 3a, b) in the studied material measuring ca. 665 µm in diameter and enveloped by a smooth, thin external layer (chorion membrane or egg capsule?) which probably has phosphatized organic matter (blastomeres?) in its interior. Although the preserved features are insufficient for conclusive determination, it is probable that the specimen may represent a fossilized scalidophoran worm embryo *Markuelia* known from the lower Cambrian of Siberia, middle Cambrian of China and Australia, upper Cambrian of China, and lowest Ordovician of the United States [37]. The stratigraphically youngest single fossilized embryo known previously is from the lower Tremadocian (Lower Ordovician) of Nevada [37]; however, this embryo was revealed from the flat-pebble conglomerate, and the specimen might be re-deposited from the underlying strata of Cambrian age. If the suggested affinity of the specimen from the Fenxiang Formation is correct then it appears to represent geologically youngest and exceedingly rare finding of cleavage stage embryo with preserved blastomeres (Fig. 3a; compare with Dong et al. [38], Fig. 1b).

In a few acetic acid residua obtained from the limestone samples, several phosphatic tubular specimens have been found (Fig. 3h–k). These tubes, although fragmentarily preserved, attain up to about 2 mm in length and 65 µm–420 µm in diameter and have longitudinal rows of minute setae. These setae, or blisters, are basally set into elliptical sockets and most probably were slightly movable and thus could allow mechanosensory transduction from the environment to the nerve cells. The sockets are well visible in case the setae are broken off, sometimes revealing longitudinal phosphatic filaments (phosphatized infesting bacteria?) deep in the socket opening (Fig. 3i, j). It seems that the tubular specimens represent arthropod appendages or furca.

## Conclusions

The preliminary results of our research briefly reviewed in this paper indicate that more extensive paleontological works on the Fenxiang Formation may significantly change the current picture of the Ordovician world. Discovery of new soft-bodied and skeletonized fossils in the Early Ordovician of China contributes new important data on the prelude of one of the greatest-ever diversifications of life known as the GOBE which was initiated in the late Early Ordovician. The first metazoan reefs which appeared in the Fenxiang Formation [20, 39] created a wide range of new ecological niches and opportunities for such diversification of various animal groups culminating during the GOBE. It is worthy to note that the Fenxiang fauna, although not unique in this respect, consists of variously advanced evolutionary groups which were members of three so-called Evolutionary Faunas (EFs; Fig. 1) [40]. The linguloid brachiopods and the fossil scalidophoran embryo shown in this paper were components of the gradually decreasing Cambrian EF, while co-occurring conodonts, bryozoans, *Sphenothallus*, conulariid, and graptolites were members of the Paleozoic EF which acquired during Ordovician a dominant position in the Paleozoic marine ecosystems. Discovery of advanced hydrozoan colony, antipatharians, and nematode traces among the Fenxiang fossils has shown that forerunners of the Modern EF were also represented at that time.
